# Prehabilitative parenteral nutrition and surgery-related muscle loss in patients undergoing major upper gastrointestinal surgery: a single-centre observational cohort study

**DOI:** 10.3389/fnut.2026.1933021

**Published:** 2026-09-09

**Authors:** Natasia Nicole Deser, Hans Alexander Mahendran, Guo Hou Loo, Nik Ritza Kosai

**Affiliations:** 1 Upper Gastrointestinal Surgery Unit, Department of Surgery, Hospital Sultanah Aminah, Johor Bahru, Johor, Malaysia; 2 Upper Gastrointestinal, Bariatric and Metabolic Surgery Unit, Department of Surgery, Faculty of Medicine, Universiti Kebangsaan Malaysia, Kuala Lumpur, Malaysia

**Keywords:** bioimpedance analysis, parenteral nutrition, phase angle, prehabilitation, skeletal muscle mass, surgery-related muscle loss, upper gastrointestinal surgery

## Abstract

**Introduction:**

Surgery-related muscle loss (SRML) is common after major upper gastrointestinal (GI) surgery and is associated with worse recovery and survival, and prehabilitative parenteral nutrition (PN) has been proposed to attenuate it, yet data from Southeast Asian populations are absent. We set out to characterise perioperative muscle change and to test whether the amount of protein and energy delivered per kilogram body weight was associated with muscle preservation.

**Methods:**

We conducted a single-centre observational cohort study of 115 adults who underwent elective major upper GI surgery at Hospital Sultanah Aminah, Johor Bahru, and received 800 kcal/day premixed prehabilitative PN (38 g amino acids), as a supplement to oral or enteral intake, for 7 days before surgery. Body composition was measured by segmental bioimpedance analysis (InBody 770) at admission and on postoperative days (PODs) 3, 7, 30, and 90. The primary outcome was clinically relevant SRML, defined as a decrease in skeletal muscle mass (SMM) of 10% or more from admission to POD 7; 111 patients had an evaluable POD 7 measurement.

**Results:**

Patients had a median age of 61 years, 58.3% were male, 70.4% had malignant disease, and 19.1% were underweight; median total protein delivery (PN plus oral or enteral intake) was 1.4 g/kg/day. From admission to POD 7, SMM decreased by a mean of 2.6%, phase angle by 6.2% and body mass index by 3.1% (all *p* < 0.001). Clinically relevant SRML occurred in 18 of 111 evaluable patients (16.2%). Protein delivered per kilogram was not associated with the change in SMM (Spearman rho = 0.05, *p* = 0.59), and energy per kilogram showed only a weak, non-significant trend (Spearman rho = 0.16, *p* = 0.09). In exploratory multivariable models limited by only 18 events, only a higher baseline SMM predicted greater relative loss, and no delivery variable predicted SRML.

**Discussion:**

In a real-world prehabilitative PN cohort, clinically relevant muscle loss was relatively infrequent, but protein delivery was not independently associated with muscle preservation, consistent with anabolic resistance during surgical stress. The single-arm design precludes causal inference, and the narrow range of protein exposure may have left the study underpowered to detect a dose-response relationship. Controlled studies with contemporaneous non-PN comparators, wider exposure contrasts and functional endpoints are required.

## 1 Introduction

Major upper gastrointestinal (GI) surgery imposes a profound metabolic stress that disrupts inflammatory, hormonal and genomic homeostasis. The resulting hypermetabolic and hypercatabolic state drives skeletal muscle wasting, impaired immunity and delayed wound healing, and in its severe form contributes to organ dysfunction and death (1). The postoperative depletion of muscle mass is captured clinically as surgery-related muscle loss (SRML), a decline in muscle mass that follows major surgical trauma (1, 2). A widely used operational threshold defines clinically relevant SRML as a loss of 10% or more of skeletal muscle within 1 week of surgery (2).

SRML is not a benign phenomenon. In a prospective cohort of patients undergoing major abdominal cancer surgery, 39% developed clinically relevant SRML within 1 week, and this loss was independently associated with insufficient postoperative nutritional intake and physical activity and with reduced one-year survival (2). Related work has linked acute perioperative muscle wasting to postoperative complications, prolonged hospitalisation, higher costs, readmission and impaired quality of life, with older patients being particularly vulnerable because of a blunted capacity for muscle protein synthesis (3, 4).

Muscle mass can be quantified by dual-energy X-ray absorptiometry, computed tomography or magnetic resonance imaging, but these methods are costly, are not readily repeatable at the bedside and may involve ionising radiation (5). Segmental multifrequency bioimpedance analysis (BIA) offers a rapid, non-invasive and repeatable alternative that estimates skeletal muscle mass (SMM), lean body mass (LBM) and phase angle, the last being an index of cell membrane integrity and cellular health that carries prognostic value in surgical and oncological populations.

Nutritional optimisation before surgery is a rational strategy to build metabolic reserve and offset the catabolic response to operation (6, 7). Preoperative parenteral nutrition (PN) can replete acute deficits, deliver a consistent supply of macronutrients and micronutrients and, in experimental and clinical work, promote muscle protein synthesis, attenuate autophagy and enhance immune function (8–11). Guidelines recommend an energy target in the region of 25–30 kcal/kg/day and a protein target of 1.5 g/kg/day for surgical patients, and preoperative nutritional support is generally advised for at least 7 days when malnutrition is present (12–14). Premixed, commercially standardised PN has an acceptable safety profile and a lower risk of bloodstream infection than compounded preparations (15, 16), while the principal early hazard, refeeding syndrome, is mitigated by structured monitoring (17).

Prehabilitation, defined as the enhancement of functional capacity so that a patient can better withstand the stress of surgery (18), has most often combined exercise with nutrition. Trials of combined prehabilitation in oesophagogastric cancer surgery have improved functional capacity without consistently altering complications or length of stay (19), and higher energy and protein provision has been associated with improved outcomes in malnourished critically ill patients (20, 21). At Hospital Sultanah Aminah, Johor Bahru (HSAJB), prehabilitative PN is embedded in the Upper GI Unit protocol: patients scheduled for major upper GI surgery are admitted 7 days early, undergo baseline BIA and receive 800 kcal/day supplementary premixed PN in addition to their oral or enteral intake. Despite the routine use of this protocol, its effect on SRML had not been formally evaluated, and no data on prehabilitative PN and SRML exist for Malaysia or the wider Southeast Asian region.

We therefore conducted this study with two aims. The primary objective was to determine whether the amount of protein delivered per kilogram body weight was associated with the change in muscle mass across major upper GI surgery in patients receiving prehabilitative PN. The secondary objective was to relate protein and energy delivery to changes in other body composition indices, namely LBM, phase angle and body weight, and to describe the incidence and trajectory of clinically relevant SRML.

## 2 Materials and methods

### 2.1 Study design and setting

This was a single-centre observational cohort study conducted at the Upper Gastrointestinal Surgery Unit, Hospital Sultanah Aminah, Johor Bahru, Malaysia. The study was reported in accordance with the STROBE recommendations for observational research.

### 2.2 Participants

Consecutive adults (aged over 18 years) who underwent elective major upper GI surgery between October 2023 and June 2025, and who received prehabilitative PN as part of the unit protocol, were eligible. Patients were excluded if the observation period was shorter than 90 days, if no BIA measurement was available on postoperative day (POD) 7, if fluid balance was expected to confound bioimpedance (end-stage renal failure, congestive cardiac failure or renal replacement therapy), or if they had intestinal failure. Universal (census) sampling was used, and no *a priori* selection beyond these criteria was applied.

### 2.3 Prehabilitative parenteral nutrition protocol

Eligible patients were admitted 7 days before the planned operation. A dietitian reviewed each patient and calculated individual requirements. In addition to oral or enteral intake, patients received 800 kcal/day premixed PN through a peripheral line for the seven preoperative days, and were monitored for catheter-related sepsis and for biochemical features of refeeding. Total energy and protein delivered during the prehabilitation window were recorded and expressed both as absolute daily amounts and as amounts per kilogram of admission body weight. The PN component was identical for every patient and consisted of one 1,206 mL bag per day of a commercially premixed three-chamber peripheral formulation (SmofKabiven Peripheral, Fresenius Kabi), delivering 800 kcal of total energy (700 kcal of non-protein energy), 38 g of amino acids (6.2 g of nitrogen, equivalent to approximately 39 g of protein), 85 g of dextrose and 34 g of lipid, at an osmolarity of approximately 850 mOsm/L. Oral or enteral intake accounted for the remainder of the recorded totals, so all between-patient variation in energy and protein delivery arose from oral or enteral intake rather than from PN. Delivery variables are consequently reported throughout as total delivery, with the fixed PN contribution stated separately, and the exposure examined in this study should be understood as the total nutritional delivery achieved within a prehabilitative PN pathway rather than as a variable dose of PN itself.

### 2.4 Body composition assessment

Body composition was measured using a segmental multifrequency bioimpedance analyser (InBody 770) at admission (preoperative baseline) and on POD 3, 7, 30 and 90. Recorded variables were body weight, LBM, SMM and phase angle. Measurements were taken according to the manufacturer’s standard protocol. Standing height was measured at baseline, and body mass index (BMI, weight in kilograms divided by height in metres squared) and a height-normalised skeletal muscle index (SMM divided by height in metres squared) were derived at each timepoint. Bioimpedance infers lean tissue from the conductive properties of body water and is therefore sensitive to hydration status and to the distribution of water between the intracellular and extracellular compartments. Cumulative perioperative fluid balance, intraoperative and postoperative fluid administration and formal clinical assessment of oedema were not recorded prospectively in this cohort and were therefore unavailable for covariate adjustment or for sensitivity analysis; this constraint is considered in detail in the Discussion.

### 2.5 Outcomes and definitions

The primary outcome was clinically relevant SRML, defined *a priori* as a decrease of 10% or more in SMM from admission to POD 7 (2). Secondary outcomes were the relative changes from admission to POD 7 in LBM, phase angle and body weight, and the longitudinal trajectory of these indices to POD 30 and POD 90. The prespecified primary analytic objective was the association between protein delivered per kilogram and the relative change in SMM.

### 2.6 Data handling and missing data

Measurements that were not obtained at a given timepoint were denoted in the dataset by numeric sentinel codes (99999) and were recoded as missing before analysis. Complete admission data were available for all 115 patients. During verification of the dataset for this revision, two patients were found to have a recorded lean body mass identical to their skeletal muscle mass at admission (and, in one of the two, also at POD 3). Because skeletal muscle mass is a component of lean body mass, these values are not physiologically possible and represent a transcription error in which the skeletal muscle mass value was entered into the lean body mass field. The affected lean body mass values were recoded as missing; the skeletal muscle mass, weight and phase angle data for these patients are unaffected and were retained. Analyses involving lean body mass are therefore based on 113 patients at admission and 109 at POD 3 and POD 7, and the denominator is stated wherever it differs from the primary analytic sample. Serial measurements were missing for 4 patients at POD 3, 4 at POD 7 (evaluable *n* = 111), 9 at POD 30 and 10 at POD 90. All longitudinal analyses used available-case data, with the denominator reported at each timepoint. Multiple imputation was considered but not undertaken. The missing values were confined to the serial body composition outcomes rather than to covariates, affected between 3.5 and 8.7% of observations depending on the timepoint, and were not accompanied by auxiliary variables that would have made imputation informative; under these conditions imputing the outcome would have imposed additional modelling assumptions without an appreciable gain in precision (22). Available-case analysis was therefore used throughout, and the reliance on it is acknowledged as a limitation.

### 2.7 Sample size

The recruitment target was set *a priori* with reference to the events-per-variable principle of Peduzzi et al. (23). Eight candidate prognostic variables were specified at the design stage (age, sex, disease type, body mass index, handgrip strength, phase angle, skeletal muscle mass and lean body mass). SRML was listed alongside these variables in the study protocol, but it is the outcome rather than a prognostic variable and is not treated as one here. Applying a requirement of ten patients per candidate variable yielded a minimum of 90 patients, and allowing for a 20% dropout rate raised the recruitment target to 108 patients; 115 patients were available for analysis.

Two departures from this plan require explicit acknowledgement. First, handgrip strength was not collected during the study period and could not be entered into any model. Second, and more consequentially, the calculation was framed in terms of patients per candidate variable rather than events per variable. Only 18 patients met the criterion for clinically relevant SRML, so the logistic regression model containing six covariates has an events-per-variable ratio of approximately three. This is well below the conventional threshold of ten (23) and below the range within which relaxation of that threshold is generally considered defensible (24), and the resulting coefficients are liable to bias and instability. The logistic model is therefore presented as exploratory and hypothesis-generating. The study is adequately sized to estimate the incidence of clinically relevant SRML and to characterise body composition trajectories, but it is not sized to identify independent predictors of the binary endpoint, and the multivariable results should be read accordingly.

### 2.8 Statistical analysis

Continuous variables are summarised as mean with standard deviation or median with interquartile range (IQR) according to distribution, and categorical variables as counts and percentages. The normality of paired differences was assessed with the Shapiro–Wilk test; within-patient change from admission to POD 7 was tested with the paired *t* test or the Wilcoxon signed-rank test as appropriate. The association between nutritional delivery and the relative change in each body composition index was examined with Pearson and Spearman correlation. A multivariable linear regression model for the relative change in SMM to POD 7 included protein per kilogram, energy per kilogram, age, sex, malignancy and baseline SMM; a sensitivity model substituted baseline BMI for baseline SMM. Predictors of clinically relevant SRML were examined with logistic regression including the same covariates with baseline phase angle in place of baseline SMM. Covariates were fixed in advance rather than selected from the data. As set out in section 2.7, however, the events-per-variable ratio achieved for the binary endpoint was approximately three, and both multivariable models are therefore reported as exploratory and hypothesis-generating rather than confirmatory. Model performance is summarised by the coefficient of determination for the linear model and by the area under the receiver operating characteristic curve (*c*-statistic) for the logistic model. No adjustment was possible for the type or extent of the operation, operative duration or blood loss, cumulative perioperative fluid balance, postoperative complications, intensive care unit admission, the timing and intensity of mobilisation, or actual nutritional intake after the prehabilitation window, because these variables were not captured in the study dataset. The incidence of SRML was compared across protein adequacy (1.5 g/kg/day or more versus below) and pathology using the Fisher exact test. A two-sided *p*-value below 0.05 was considered significant. Analyses were performed in Python 3 (pandas, SciPy and statsmodels).

### 2.9 Ethics

The study was conducted in accordance with the Declaration of Helsinki and the Malaysian Good Clinical Practice Guideline, and was approved by the Research Ethics Committee, Universiti Kebangsaan Malaysia (JEP-2024-507) and the Medical Research and Ethics Committee, Ministry of Health Malaysia (NMRR ID-25-03464-4VQ). Written informed consent was obtained from all participants. Further details are provided in the Ethics Statement.

## 3 Results

### 3.1 Baseline characteristics and nutritional delivery

A total of 115 patients were analysed. The median age was 61 years (IQR 50–67, full range 21–86); 67 patients (58.3%) were male, and 81 (70.4%) had malignant disease. At admission, median body weight was 56.9 kg (IQR 47.6–64.7), median SMM 21.8 kg (IQR 18.4–26.5), median LBM 38.6 kg (IQR 32.6–45.6; *n* = 113) and median phase angle 4.3 degrees (IQR 3.7–4.8). Median height was 163 cm (IQR 156–167) and median BMI was 21.5 kg/m^2^ (IQR 19.5–24.1); 22 patients (19.1%) were underweight (BMI below 18.5 kg/m^2^) and 22 (19.1%) were overweight or obese (BMI 25 kg/m^2^ or above), underscoring the nutritional vulnerability of the cohort. The median baseline skeletal muscle index was 8.5 kg/m^2^ (IQR 7.5–9.7), 9.3 kg/m^2^ in men and 7.5 kg/m^2^ in women. During the seven-day prehabilitation window, patients received a median of 1980 kcal/day (IQR 1600–2,408) and 75.0 g/day of protein (IQR 64.5–89.6), corresponding to 36.2 kcal/kg/day (IQR 30.3–44.6) and 1.4 g/kg/day of protein (IQR 1.1–1.7). Fifty patients (43.5%) met the guideline protein target of 1.5 g/kg/day or more. These figures describe total delivery. The PN component was fixed at 800 kcal and 38 g of amino acids per day for every patient, corresponding to a median of 14.1 kcal/kg/day (IQR 12.4–16.8) and 0.67 g/kg/day (IQR 0.59–0.80). PN therefore supplied a median of 40.4% (IQR 33.2–50.0) of the total energy and 50.7% (IQR 42.4–58.9) of the total protein delivered. The remainder came from oral or enteral intake, which contributed a median of 21.2 kcal/kg/day (IQR 16.0–29.4) and 0.73 g/kg/day (IQR 0.46–0.96), and which accounts for all of the observed between-patient variation in delivery. Baseline characteristics are shown in Table 1.

**Table 1:** Baseline characteristics and prehabilitation nutritional delivery.

Characteristic	Value (*N* = 115)
Age, years, median (IQR)	61 (50–67)
Age, years, mean (SD)	57.6 (14.4)
Male sex, *n* (%)	67 (58.3)
Malignant disease, *n* (%)	81 (70.4)
Height, cm, median (IQR)	163 (156–167)
Body weight, kg, median (IQR)	56.9 (47.6–64.7)
Body mass index, kg/m^2^, median (IQR)	21.5 (19.5–24.1)
Underweight (BMI < 18.5), *n* (%)	22 (19.1)
Overweight or obese (BMI ≥ 25), *n* (%)	22 (19.1)
Skeletal muscle mass, kg, median (IQR)	21.8 (18.4–26.5)
Skeletal muscle index, kg/m^2^, median (IQR)	8.5 (7.5–9.7)
Lean body mass, kg, median (IQR), *n* = 113	38.6 (32.6–45.6)
Phase angle, degrees, median (IQR)	4.3 (3.7–4.8)
Total energy delivered, kcal/day, median (IQR)	1980 (1,600–2,408)
Total protein delivered, g/day, median (IQR)	75.0 (64.5–89.6)
Energy delivered, kcal/kg/day, median (IQR)	36.2 (30.3–44.6)
Protein delivered, g/kg/day, median (IQR)	1.4 (1.1–1.7)
Protein ≥ 1.5 g/kg/day, *n* (%)	50 (43.5)

### 3.2 Body composition change and incidence of SRML

**Figure 1:**
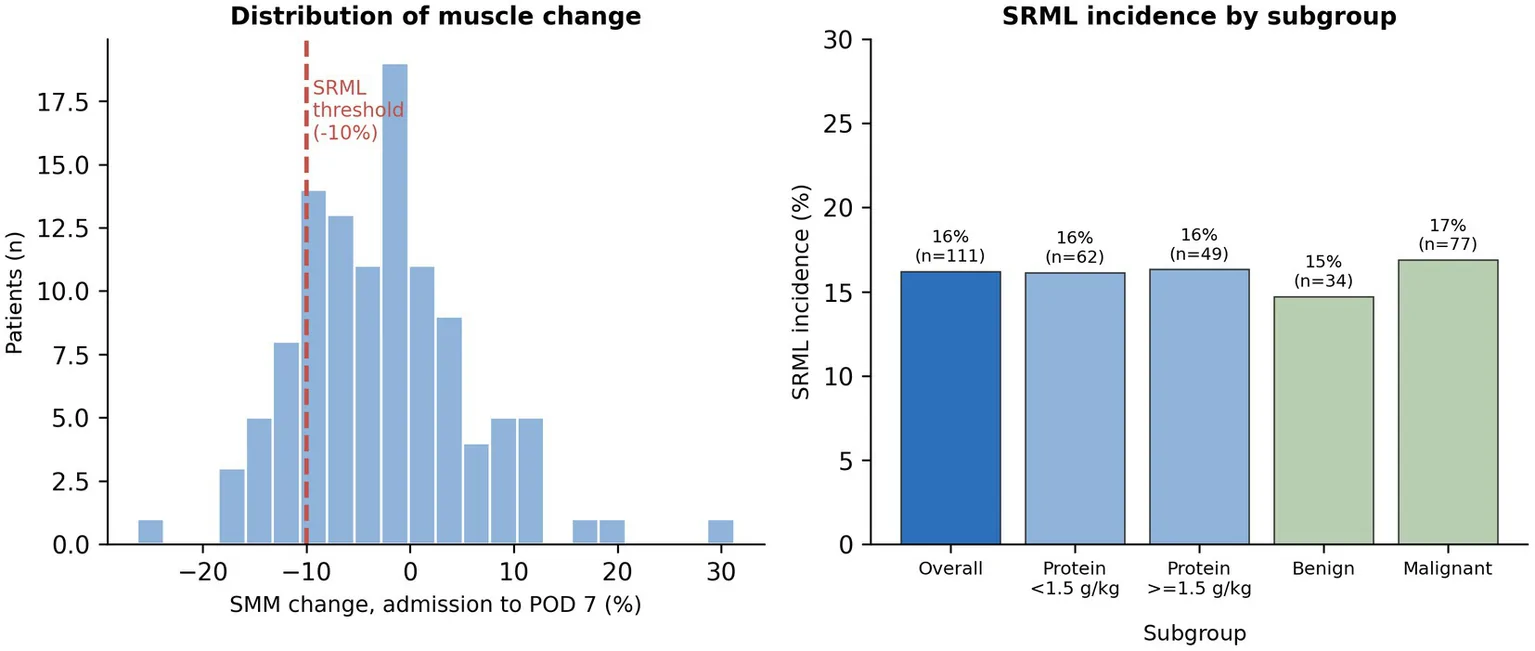
Left, distribution of relative skeletal muscle mass change from admission to POD 7 with the SRML threshold. Right, incidence of clinically relevant SRML overall and by protein adequacy and pathology, with subgroup sizes. Both panels are based on the 111 patients with an evaluable POD 7 measurement, of whom 18 met the criterion for clinically relevant SRML.

Muscle loss was already detectable by POD 3 and deepened by POD 7. By POD 3 (*n* = 111), SMM had fallen by a mean of 1.6%, LBM by 1.2% (*n* = 109), phase angle by 5.6% and body weight by 1.5% (all Wilcoxon *p* < 0.001). From admission to POD 7 (evaluable *n* = 111), all indices declined further: mean SMM fell by 2.6% (from 22.31 to 21.61 kg; paired *t* test *p* < 0.001), LBM by 2.4% (*n* = 109; Wilcoxon *p* < 0.001), phase angle by 6.2% (Wilcoxon *p* < 0.001), body weight by 3.1% and BMI by 3.1% (both Wilcoxon *p* < 0.001). The paired changes to POD 7 are summarised in Table 2. Clinically relevant SRML, a loss of 10% or more of SMM by POD 7, occurred in 18 of 111 patients (16.2%, 95% CI 10.5–24.2). The distribution of relative SMM change and the SRML threshold are shown in Figure 1.

**Table 2:** Change in body composition from admission to postoperative day 7 (*n* = 111; *n* = 109 for lean body mass).

Index	Admission	POD 7	Mean change	Relative change	*p* value
Skeletal muscle mass, kg	22.31	21.61	−0.70	−2.6%	<0.001
Lean body mass, kg	39.13	38.01	−1.12	−2.4%	<0.001
Phase angle, degrees	4.26	3.99	−0.27	−6.2%	<0.001
Body weight, kg	57.11	55.10	−2.01	−3.1%	<0.001

Relative change is the mean of within-patient percentage changes. Because this is the mean of individual percentage changes rather than the ratio of the mean absolute change to the mean baseline value, the two quantities do not coincide; the corresponding ratio-of-means values are −3.1% for skeletal muscle mass, −2.9% for lean body mass, −6.4% for phase angle and −3.5% for body weight. Both estimands are given so that the relationship between the absolute and relative columns is unambiguous.

### 3.3 Association of nutritional delivery with muscle change

**Figure 2:**
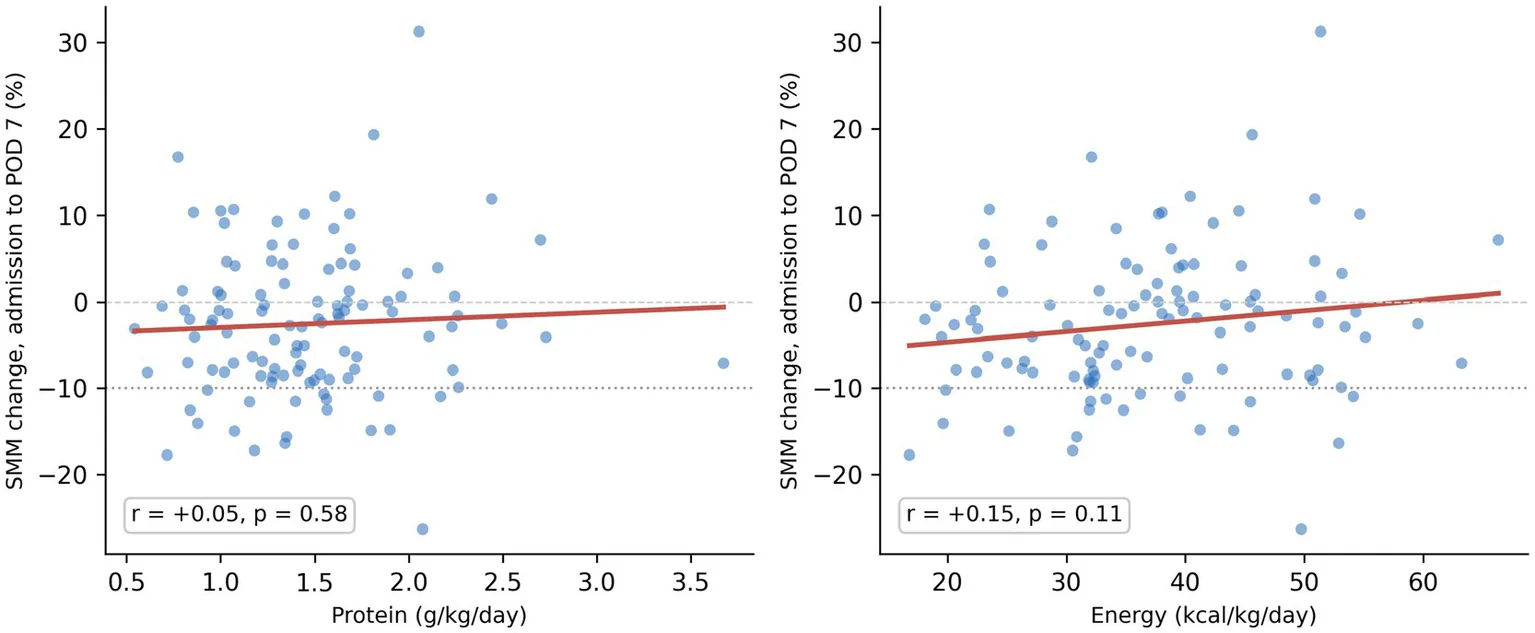
Relationship between weight-indexed nutritional delivery and relative change in skeletal muscle mass from admission to postoperative day 7. Left, protein per kilogram; Right, energy per kilogram. The dotted horizontal line marks the 10% loss threshold for clinically relevant SRML; the solid line is the least-squares fit. Coefficients annotated in each panel are Pearson correlation coefficients; the corresponding Spearman coefficients are reported in Table 3. Both panels include the 111 patients with an evaluable POD 7 measurement.

For the primary objective, the amount of protein delivered per kilogram was not associated with the relative change in SMM to POD 7 (Pearson *r* = 0.05, *p* = 0.58; Spearman rho = 0.05, *p* = 0.59). Energy delivered per kilogram showed only a weak, non-significant positive trend (Spearman rho = 0.16, *p* = 0.09). Absolute (non-weight-indexed) protein and energy delivery were not associated with SMM change. Among the secondary indices, the associations reaching significance involved BMI change (which by construction mirrors weight change, height being fixed): both energy per kilogram (rho = 0.24, *p* = 0.01) and protein per kilogram (rho = 0.19, *p* = 0.05) were weakly and positively related to BMI change, indicating that greater delivery tracked less weight and BMI loss but not muscle-specific preservation. No delivery variable was associated with the change in phase angle. Correlations are presented in Table 3, and the protein and energy dose–response relationships for SMM are shown in Figure 2.

**Table 3:** Spearman correlation coefficients (*p*-values) between weight-indexed nutritional delivery and relative change in body composition indices from admission to POD 7 (*n* = 111; *n* = 109 for the lean body mass column).

Delivery variable	SMM change	LBM change	Phase angle change	BMI change
Protein, g/kg/day	0.05 (0.59)	0.06 (0.51)	−0.10 (0.31)	0.19 (0.05)
Energy, kcal/kg/day	0.16 (0.09)	0.18 (0.06)	0.04 (0.64)	0.24 (0.01)

### 3.4 Multivariable models

In the multivariable linear regression model for relative SMM change to POD 7 (*n* = 111), neither protein per kilogram (beta −3.01, *p* = 0.20) nor energy per kilogram (beta 0.08, *p* = 0.51) was an independent predictor after adjustment. A higher baseline SMM was the only significant covariate and predicted a greater relative loss (beta −0.61 per kg, *p* = 0.02), while age, sex and malignancy were not significant (model *R*^2^ = 0.10). Substituting baseline BMI for baseline SMM in a sensitivity model left the conclusions unchanged: no delivery variable was significant, and baseline BMI itself was not an independent predictor (*p* = 0.11). The model accounted for only a small fraction of the variability in relative SMM change (*R*^2^ = 0.10), indicating that the measured covariates leave most of the between-patient variation unexplained. The full model is shown in Table 4.

**Table 4:** Multivariable linear regression for relative change in skeletal muscle mass, admission to POD 7 (*n* = 111; *R*^2^ = 0.10; exploratory model).

Predictor	Coefficient	95% CI	*p* value
Protein, g/kg/day	−3.01	−7.65 to 1.63	0.20
Energy, kcal/kg/day	0.08	−0.15 to 0.31	0.51
Age, years	−0.08	−0.21 to 0.05	0.21
Male sex	1.83	−3.14 to 6.81	0.47
Malignant disease	0.50	−3.35 to 4.35	0.80
Baseline SMM, kg	−0.61	−1.12 to −0.09	0.02

In the logistic regression model for clinically relevant SRML (*n* = 111), no covariate reached significance, including protein per kilogram (odds ratio 0.99, 95% CI 0.21–4.70) and energy per kilogram (odds ratio 0.98, 95% CI 0.91–1.05); the model is shown in Table 5. Consistent with this, the incidence of SRML did not differ between patients meeting the 1.5 g/kg/day protein target and those below it (16.3% versus 16.1%; Fisher *p* = 1.00), nor between malignant and benign disease (16.9% versus 14.7%; Fisher *p* = 1.00). Discrimination of the logistic model was poor (*c*-statistic 0.56, barely better than the 0.50 expected by chance). With 18 events distributed across six covariates, these estimates are imprecise and unstable, and they are reported as exploratory rather than as evidence of the absence of an association. SRML incidence by subgroup is shown in Figure 1.

**Table 5:** Exploratory logistic regression for clinically relevant surgery-related muscle loss at POD 7 (*n* = 111; 18 events).

Predictor	Odds ratio	95% CI	*p* value
Protein, g/kg/day	0.99	0.21–4.70	0.99
Energy, kcal/kg/day	0.98	0.91–1.05	0.58
Age, years	1.00	0.96–1.05	0.94
Male sex	1.10	0.33–3.63	0.87
Malignant disease	1.15	0.32–4.09	0.83
Baseline phase angle	0.85	0.38–1.90	0.70

### 3.5 Longitudinal trajectory

**Figure 3:**
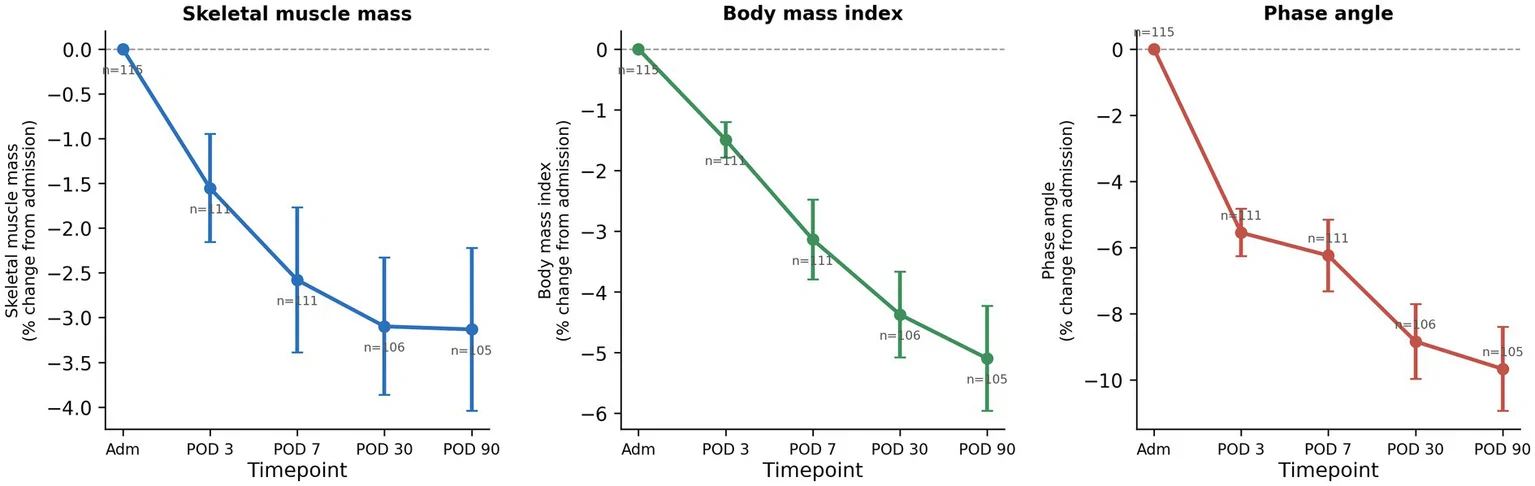
Trajectory of skeletal muscle mass (left), body mass index (centre), and phase angle (right) expressed as mean percentage change from admission, with standard error bars. Available-case numbers are shown at each time point. Skeletal muscle mass stabilises after the first postoperative month, whereas body mass index and phase angle continue to decline to day 90.

Across the follow-up period, the mean relative SMM deficit from admission deepened from 1.6% at POD 3 (*n* = 111) to 2.6% at POD 7 (*n* = 111) and 3.1% at POD 30 (*n* = 106), and was 3.1% at POD 90 (*n* = 105), indicating that muscle mass reached a nadir in the first postoperative month and did not return to baseline by 3 months. BMI, in contrast, continued to decline throughout follow-up, falling by 1.5% at POD 3, 3.1% at POD 7, 4.4% at POD 30 and 5.1% at POD 90, so that overall body mass kept contracting even after skeletal muscle mass had stabilised. Among the 18 patients with SRML at POD 7 (mean SMM deficit 14.1%), the deficit narrowed to 8.0% at POD 30 and 6.4% at POD 90, consistent with partial recovery. Phase angle, in contrast, declined progressively, from 5.6% below baseline at POD 3 to 6.2% at POD 7, 8.8% at POD 30 and 9.7% at POD 90, suggesting an ongoing decline in cellular integrity that outlasted the recovery of muscle quantity. The trajectories of SMM, BMI and phase angle are shown in Figure 3.

## 4 Discussion

In this first Southeast Asian cohort of patients receiving standardised prehabilitative PN before major upper GI surgery, three findings stand out. First, clinically relevant SRML at 1 week occurred in 16.2% of patients, numerically lower than the 39% reported in a cohort of major abdominal cancer surgery managed without a structured prehabilitation nutrition protocol (2), although causal inference regarding the effect of prehabilitative PN cannot be made in the absence of a contemporaneous control group. Second, and contrary to the study hypothesis, the amount of protein delivered per kilogram was not associated with muscle preservation, whether examined by correlation, by multivariable regression or by comparison across the guideline protein threshold. Third, phase angle continued to fall to POD 90 even as muscle quantity partially recovered, pointing to a persistent deficit in cellular quality that a mass-based endpoint does not capture.

The relatively low incidence of clinically relevant SRML is encouraging and is compatible with a protective contribution from prehabilitative nutrition, but the single-arm design does not allow that inference to be drawn with confidence. Because every patient in the cohort received PN, the difference from published figures could equally be attributable to differences in baseline patient characteristics, to differences in the mix and magnitude of the operations performed, to differences in perioperative management, or to differences in the measurement modality and threshold used to define muscle loss. The comparison is therefore hypothesis-generating and should not be read as an estimate of treatment effect. Incidence estimates for SRML are sensitive to the measurement modality and threshold used: cohorts defining SRML by ultrasound cross-sectional area or by a 5% threshold report higher rates than those using a 10% BIA-based threshold, so part of the difference from historical figures reflects methodology rather than biology. What can be stated is that, within a protocolised prehabilitation pathway, most patients preserved the large majority of their skeletal muscle across the first postoperative week.

The absence of a protein dose–response relationship is the most clinically salient result. It is tempting to assume that delivering more protein will proportionally spare muscle, but our data do not support that assumption in the perioperative window. This is biologically coherent. Surgical stress produces a state of anabolic resistance in which the muscle protein synthetic response to amino acid provision is blunted, and in which inflammatory and neuroendocrine signalling drives proteolysis largely independent of substrate supply (1, 25, 26). In that setting the rate-limiting factor for muscle preservation is not the quantity of protein presented but the capacity of muscle to use it, which is modulated by inflammation, immobilisation, insulin resistance and the magnitude of the operative insult. Energy per kilogram, rather than protein per kilogram, showed the numerically larger association with muscle change, but this did not reach significance and is at most hypothesis-generating, consistent with a permissive role for adequate total energy in limiting the diversion of amino acids to gluconeogenesis. The finding that higher baseline SMM predicted greater relative loss most likely reflects a combination of regression toward the mean and the simple fact that patients with more muscle have more to lose.

An important caveat concerns the range of protein exposure that was actually observed. Because the PN component was fixed and the whole cohort was managed under a single unit protocol, protein delivery clustered tightly around a median of 1.4 g/kg/day with an interquartile range of only 1.1–1.7 g/kg/day, and fewer than half of patients reached the guideline target of 1.5 g/kg/day. Half of that protein came from the PN itself, which was fixed at 0.67 g/kg/day (IQR 0.59–0.80) and therefore contributed no variance at all; the only variable component was oral or enteral intake, at a median of 0.73 g/kg/day (IQR 0.46–0.96). A null result across so narrow an exposure band cannot exclude a dose–response relationship that would become apparent over a wider range, and in that respect the study was constrained by the homogeneity of the exposure as much as by the size of the cohort. The appropriate reading of our data is therefore not that protein delivery is irrelevant to muscle preservation, but that variation within the range achievable under routine care did not detectably influence it. That reading is consistent with randomised evidence from critical illness, in which deliberately widening the protein exposure contrast did not shorten time to discharge alive (27), and with mechanistic work showing that acute muscle wasting in catabolic states is driven predominantly by depressed protein synthesis and accelerated breakdown rather than by limitation of substrate (26).

A parallel dissociation was seen between body mass and muscle. BMI continued to fall to 3 months while SMM had already stabilised after the first postoperative month, implying that the later weight loss reflected fat and fluid rather than a further decline in skeletal muscle, and reinforcing that a weight or BMI endpoint alone is an unreliable surrogate for muscle preservation in this setting. Nearly one in five patients was underweight at admission, confirming the malnourished profile that motivates a prehabilitation nutrition pathway in the first place. The progressive decline in phase angle deserves emphasis. Phase angle reflects the integrity and function of cell membranes rather than the bulk of tissue, and its continued fall to 3 months, at a time when SMM had partially recovered, suggests that recovery of muscle mass may precede recovery of muscle quality. If confirmed, this dissociation would argue for incorporating a quality metric such as phase angle, alongside mass, into the assessment of perioperative nutritional interventions, since an endpoint restricted to mass may overstate functional recovery.

The dependence of the primary endpoint on bioimpedance warrants fuller emphasis than a routine caveat. Because BIA infers lean tissue from the conductive properties of body water, any perturbation of hydration, or of the partition of water between the intracellular and extracellular compartments, will alter the estimate independently of true muscle protein. Major upper GI surgery perturbs precisely this. Intraoperative and resuscitative fluid loading expands the extracellular compartment, the inflammatory response shifts water outward across the cell membrane, and both effects are maximal in the first postoperative days before resolving over the following week. Serial bioimpedance in patients undergoing transthoracic oesophagectomy demonstrates this pattern directly, with extracellular water and the extracellular to total body water ratio rising sharply after operation and peaking within the first two postoperative days (28). Part of the apparent decline in SMM between POD 3 and POD 7 may therefore represent resolution of extracellular expansion rather than loss of contractile tissue; conversely, in patients who remained oedematous at POD 7, retained extracellular water would tend to mask true muscle loss and bias the estimated incidence of clinically relevant SRML downward. We could not quantify either possibility, because cumulative fluid balance, postoperative fluid administration and formal oedema assessment were not recorded prospectively, so neither covariate adjustment nor a sensitivity analysis was feasible. Excluding patients with end-stage renal failure, congestive cardiac failure or renal replacement therapy removes the most extreme sources of fluid redistribution but does not address the ordinary fluid shifts that accompany major surgery. This is the most important measurement limitation of the study, and prospective capture of fluid balance together with an oedema index such as the extracellular to total body water ratio should be regarded as a minimum requirement in any successor study.

Taken together, these results reframe the clinical question. They suggest that the value of a prehabilitation pathway may lie less in the precise protein dose than in the combination of early nutritional contact, dietitian-led assessment and the surrounding elements of care, and they caution against treating protein prescription as a standalone lever for muscle preservation. This is consistent with the trial literature on combined prehabilitation, in which multimodal programmes improve functional capacity without consistent effects on downstream complications (19).

This study has several strengths. It is the first to describe prehabilitative PN and SRML in a Southeast Asian population; it uses a protocolised intervention with consistent delivery; it follows patients to 90 days with a serial, repeatable body composition measure; and its analysis is prespecified and transparent about data quality. It also has important limitations. The design is observational and single-arm, without a contemporaneous non-PN comparator, so no causal claim about the effect of PN on SRML can be made. Postoperative BIA is influenced by perioperative fluid shifts, which can transiently inflate or deflate estimates of lean tissue in the early postoperative period and represent a source of measurement error that no bedside modality fully avoids. The analysis was also unadjusted for several variables that plausibly dominate perioperative muscle loss: the type and magnitude of the operation performed, operative duration and blood loss, cumulative perioperative fluid balance, the occurrence and severity of postoperative complications, intensive care unit admission and its duration, the timing and intensity of mobilisation and rehabilitation, and actual oral, enteral or parenteral intake in the postoperative period. None of these were captured in the study dataset. Their omission constrains the interpretation of the dose–response analyses in particular, since a patient who sustains an anastomotic leak and spends a week in intensive care will lose muscle for reasons that no achievable difference in preoperative protein delivery could offset, and such patients cannot be distinguished within our data. The dataset likewise did not include handgrip strength or a formal Subjective Global Assessment, both of which had been envisaged at the design stage, and no walking test, complication grading, length of stay or quality-of-life measure was collected. The study therefore describes body composition alone and cannot demonstrate that preserved muscle translated into greater strength, better physical performance, fewer complications, shorter hospitalisation or improved quality of life; because muscle mass matters clinically only insofar as it supports function and recovery, this materially limits the translational reach of the findings. A minority of serial measurements were missing and were handled by available-case analysis rather than by imputation. Finally, the multivariable models explained little of the observed variability (*R*^2^ = 0.10 for relative SMM change) and, with 18 events across six covariates in the logistic model, rest on an events-per-variable ratio of approximately three; they are adequate to show that no strong and simple relationship between delivery and muscle preservation is present in these data, but they cannot exclude modest associations, their coefficients should not be used for prediction, and a null association should not be read as proof of no effect.

The clearest implication for practice is that adequate protein delivery, while necessary, is unlikely to be sufficient on its own to prevent perioperative muscle loss, and that attention should extend to the modifiable drivers of anabolic resistance, including early mobilisation, control of the inflammatory response and glycaemic management. For research, the priority is a controlled study: ideally a randomised trial or, where randomisation to no supplementary nutritional support is not acceptable, a prospective study with a well-matched contemporaneous comparator managed without supplementary PN or on a lower-intensity pathway. Such a study should widen the protein exposure contrast deliberately, for example by comparing a standard target of about 1.2 g/kg/day with a target of 2.0 g/kg/day, so that a dose–response relationship can be detected if one exists; record PN, oral and enteral intake as separate streams both before and after operation; capture cumulative fluid balance and an oedema index alongside every body composition measurement, and where feasible corroborate bioimpedance with a hydration-independent modality such as skeletal muscle area at the third lumbar vertebra on computed tomography already obtained for staging; pair morphological endpoints with functional ones, at minimum handgrip strength and a walking test, and with patient-centred clinical endpoints including complications graded by the Clavien-Dindo classification, length of stay and readmission; retain phase angle as a cellular endpoint, given the dissociation observed here; and be powered on the binary SRML endpoint itself, which for an anticipated incidence of the order of 16% implies a cohort substantially larger than the present one.

## 5 Conclusion

In a real-world cohort of patients undergoing major upper GI surgery within a standardised prehabilitative PN pathway, clinically relevant SRML at 1 week affected about one in six patients. This proportion is numerically lower than that reported in comparable cohorts without structured prehabilitation nutrition, but the absence of a contemporaneous control group precludes any causal attribution to PN and the comparison should be regarded as exploratory. However, the amount of protein delivered per kilogram did not predict muscle preservation, and no nutritional delivery variable independently predicted SRML. The progressive decline in phase angle despite partial recovery of muscle mass suggests that quality and quantity of muscle recover on different timescales. These observations are consistent with anabolic resistance during surgical stress and indicate that protein dose alone is an insufficient target, although the narrow range of protein exposure achievable under a single unit protocol means that a dose–response relationship across a wider range cannot be excluded. Controlled studies with contemporaneous comparators, wider exposure contrasts, and functional, clinical and cellular endpoints are needed to define the role of prehabilitative PN.

## Data Availability

The datasets generated and analysed during this study are not publicly available owing to restrictions relating to participant privacy and institutional data governance. De-identified data are available from the corresponding author on reasonable request. Requests should be directed to Guo Hou Loo, looguohou@ukm.edu.my.
